# Non-Coding and Regulatory RNAs as Epigenetic Remodelers of Fatty Acid Homeostasis in Cancer

**DOI:** 10.3390/cancers12102890

**Published:** 2020-10-09

**Authors:** Silvia Cruz-Gil, Lara P. Fernández, Ruth Sánchez-Martínez, Marta Gómez de Cedrón, Ana Ramírez de Molina

**Affiliations:** Laboratory of Molecular Oncology, IMDEA-Food Institute, CEI UAM + CSIC, 28049 Madrid, Spain; silvia.cruz@imdea.org (S.C.-G.); lara.fernandez@imdea.org (L.P.F.); ruth.sanchez@imdea.org (R.S.-M.)

**Keywords:** molecular biology, cancer, metabolic reprogramming, fatty acid metabolism, regulatory RNAs, non-coding RNAs

## Abstract

**Simple Summary:**

Reprogramming of energy metabolism is an emerging hallmark of cancer development. Progression in a tumor cell requires new biogenesis of fatty acids (FA) for membrane synthesis, as signaling molecules or as energy input. Here, we provide a review of fatty acid metabolism misbalance from a cancer perspective (lipids’ storage formation, their hydrolysis, extra FAs uptake, FA synthesis, FA oxidation, and finally, FA activation and desaturation) and we summarize the reported non-coding RNAs affecting these processes as new strategies to target fatty acid availability in cancer cells.

**Abstract:**

Cancer cells commonly display metabolic fluctuations. Together with the Warburg effect and the increased glutaminolysis, alterations in lipid metabolism homeostasis have been recognized as a hallmark of cancer. Highly proliferative cancer cells upregulate de novo synthesis of fatty acids (FAs) which are required to support tumor progression by exerting multiple roles including structural cell membrane composition, regulators of the intracellular redox homeostasis, ATP synthesis, intracellular cell signaling molecules, and extracellular mediators of the tumor microenvironment. Epigenetic modifications have been shown to play a crucial role in human development, but also in the initiation and progression of complex diseases. The study of epigenetic processes could help to design new integral strategies for the prevention and treatment of metabolic disorders including cancer. Herein, we first describe the main altered intracellular fatty acid processes to support cancer initiation and progression. Next, we focus on the most important regulatory and non-coding RNAs (small noncoding RNA—sncRNAs—long non-coding RNAs—lncRNAs—and other regulatory RNAs) which may target the altered fatty acids pathway in cancer.

## 1. Introduction

### Fatty Acids Metabolism Importance in Cancer Progression: Epigenetic Processes as Novel Integral Approaches for Prevention and Treatment

Lipids comprise a vast group of molecules in terms of their chemical structure: fatty acid (FA) chain length, position, and number of double bonds and backbone structure (glycerol and sphingoid bases). Lipids perform multiple biochemical functions during cancer development, which involves cell membrane composition (phosphatidylcholine (PC) and phosphatidylethanolamine (PE), sterols, sphingolipids, and lyso-phospholipids (lyso-PLs)), lipid raft formation for signaling protein recruitment, and, thus, protein–protein interactions leading to signal transduction involved in cell survival, angiogenesis, and metastatic processes, as well as energy sources [1,2,3,4]. 

The majority of these lipids contain FAs in their backbones. In these cases, lipids are named saponifiable. FAs are also essential for energy storage, membrane biogenesis, and the creation of signaling molecules. FAs could be provided by exogenous sources or be synthesized de novo. Several proliferating tumor cells prefer to synthesize FAs de novo, rather than utilizing exogenous sources like the majority of non-transformed human cells [2,5,6,7]. The point of departure for de novo FA synthesis is acetyl-CoA, which connects this route with major core metabolic pathways.

Lipid metabolism abnormalities have recently become a remarkable feature of cancer metabolism. Despite initially not being paid great attention, FA metabolism abnormalities in cancer progression have been increasingly recognized in recent years [2,4,8,9,10,11]. In this sense, epigenetic processes could help to design novel approaches for the prevention and treatment of metabolic disorders in cancer. For decades, investigation has been focused on the involvement of protein-coding genes. Nevertheless, recently, a new class of molecules, named non-coding RNAs (ncRNAs), has emerged as a master regulator in shaping cellular activity regarding cancer. They comprise both oncogenic and tumor-suppressive molecules [12]. NcRNAs can be divided into two major categories based on the size of the transcript: from 20–200 nucleotides are small non-coding RNAs (sncRNAs) and above 200 nucleotides, lncRNAs. 

LncRNAs lack the ability to be translated into protein. They perform a regulatory effect at the transcriptional and post-transcriptional level through different mechanisms involving chromatin and also working with other RNA species [13]. While lncRNAs are less studied than other ncRNAs they have been functionally linked with different cancers in humans [14]. 

Regarding the sncRNAs group, they include RNAs implicated in translation such as ribosomal RNAs (rRNAs), transfer RNAs (tRNAs), small nuclear RNAs (snRNAs), and small nucleolar RNAs (snoRNAs). As it is universally known, tRNA is in charge to decode the mRNA sequence to give rise to a protein complex and rRNA is the mechanical scaffold of the ribosome that joins tRNA and mRNA to be processed to protein. The fundamental role of both RNAs in translation makes them have a vital regulatory effect on different physiological aspects and also over disease such as cancer [15]. SnRNAs regulate splicing events of the cell nucleus. Their length is about 150 nucleotides. They are different from snoRNAs which are mainly implicated on the regulation of other small RNAs, such as rRNAs and tRNAs and snRNAs through chemical modifications. Their length is approximately 60–200 nucleotides [16]. 

**Figure 1:**
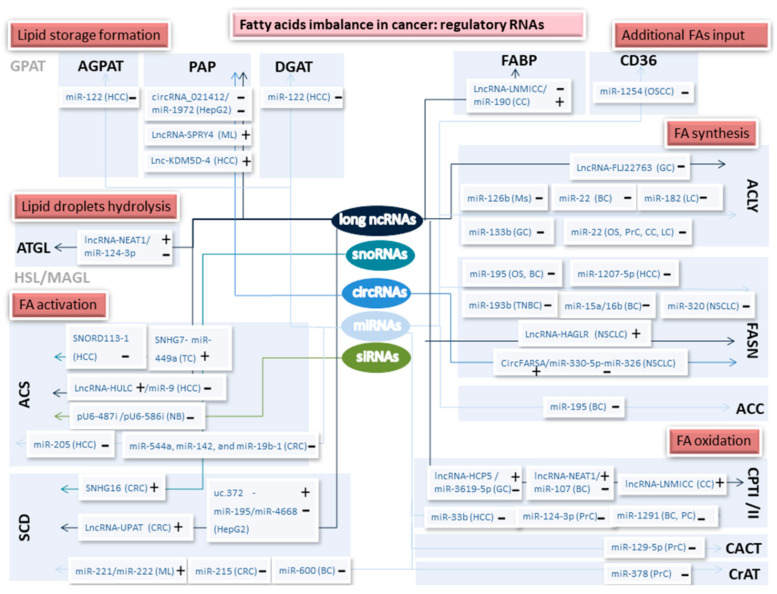
Non-coding RNAs targeting fatty acid (FA) pathways related with cancer progression.

Moreover, sncRNAs embrace also regulatory RNAs, such as short-interfering RNAs (siRNAs), microRNAs (miRNAs), tRNA derived fragments (tRF), and piwi-interacting RNAs (piRNAs) [17]. Finally, circular RNAs (circRNAs) are increasingly being understood as a class of ncRNA. siRNAs are able to degrade mRNA after transcription through complementary nucleotide regions interfering with gene expression. Synthetic siRNAs have been widely used as a therapy strategy in several cancer types [18]. Extensively described on the last years, miRNAs promote mRNA degradation or inhibit translation binding specifically to complementary sequences within mRNAs. They are formed by a single-stranded RNA of 19–25 nucleotides They are about to target 60% of the total of human genome and are described as frequently deregulated in tumors [19]. tRFs are small molecules (14–50 nucleotides) coming from the cleavage of a mature tRNA. They function as a mechanical interference between mRNAs and RNA-binding proteins. tRNAs exhibit great differences between race, sex, and disease status, and nowadays represent an increasing key focus in cancer research [20]. piRNAs (24–32 nucleotides) are linked to PIWI proteins, a subclass of argonaute proteins. They are involved in different epigenetic processes and post-transcriptional silencing. piRNAs have been described as both oncogenic and tumor-suppressive RNAs in tumor development [21]. CircRNAs are single-stranded RNA with their 3′ and 5′ ends covalently linked [22]. They operate as effective microRNA sponges, gene splicing, transcription, and translation regulators and interact with RNA-binding proteins [23,24]. As a general view, Figure 1 encapsulates the reviewed ncRNAs targeting FA metabolism pathways in this manuscript.

## 2. Central Nodes to Control Fatty Acids Imbalance in Cancer 

**Figure 2:**
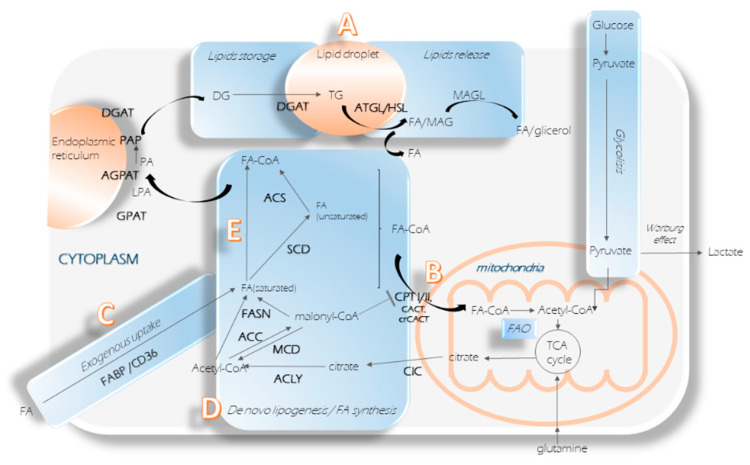
Fatty acid pathways involvement in cancer progression. (**A**) Lipid storage formation and their hydrolysis; (**B**) FA oxidation; (**C**) Extra FA input; (**D**) FA synthesis de novo; (**E**) FA activation and desaturation.

Nowadays, there is plenty of evidence suggesting that enzymes involved in the lipid synthesis pathway, and especially in FA synthesis and bioavailability, could be appealing therapeutic targets in cancer [25,26]. To this aim, five ways to target FA availability can be considered (Figure 2): targeting lipid storage formation and their hydrolysis, extra FAs uptake, FA synthesis, FA oxidation, and finally, FA activation and desaturation [4]. In the following sections we will include the study of epigenetic processes which might help to design novel approaches for the prevention and treatment of metabolic disorders in cancer, summarized in Table 1.

### 2.1. Lipids Store Formation

Lipid droplets (LDs) are organelles mainly composed of neutral lipids (triglycerides, sterol esters) able to store the majority of the energy in the cell and also crucial for different intracellular signaling pathways linked to lipid metabolism [76]. While non-transformed cells tend to rely on an exogenous source of lipids, tumor cells prefer endogenous ones. Thus, some cancer cells promote an increased generation of LDs, under both normoxia and hypoxia [77], in order to fill their requirements for new membrane biogenesis and high proliferation rates [8,78].

Their number and volume, along with the LD-associated protein ADRP expression, are significantly augmented in several cancer types [79] and are now considered as hallmarks of cancer aggressiveness [80,81]. However, these energy storage organelles present dual roles in cancer [82]. 

The glycerol–phosphate pathway is the responsible for the TG synthesis retained in the LD. Enzymes such as glycerol-3-phosphate acyltransferase (GPAT), acylglycerolphosphate acyltransferase (AGPAT), phosphatidic acid phosphohydrolase (Lipin or PAP), and diacylglycerol acyltransferase (DGAT) are part of this route, converting fatty-acyl CoA (FA–CoA) into diacylglyceride (DG) (Figure 2A, lipids storage). The pathway intermediates (lysophosphatidic acid (LPA) and phosphatidic acid (PA)) could lead to the synthesis of major structural components of biological membranes, such as different phosphoglycerides, including phosphatidylcholine (PC), phosphatidylethanolamine (PE), phosphatidylglycerol (PG), and phosphatidylserine (PS) and can act as lipid signaling molecules [83] that cancer can benefit from. 

GPATs main action is to esterify long-chain FA–CoA to glycerol 3-phosphate (G3P) producing LPA [84]. Four GPATs have been identified to date, nonetheless, little is known about their role in cancer-related processes. Recently, it has been described that the overexpression of GPAT isoform 1 endorses cell migration and poor survival in ovarian carcinoma cell lines [85]. GPAT1 deficiency in mice was also proposed to reduce susceptibility to liver tumorigenesis [86]. GPAT2 overexpression was suggested as a novel cancer-testis biomarker gene, and it also contributes to the tumor phenotype of MDA-MB-231 cells, a triple-negative breast cancer (TNBC) cell line [87]. 

The LPA is further metabolized to PA by AGPAT (LPAAT synonym), by adding an acyl group to the glycerol backbone. Eleven human AGPAT isoforms have been identified. Their overexpression has been previously associated with some human cancers, and their inhibition with cell growth arrest and death [88,89]. AGPAT1 has been recently involved in a metabolic signature able to predict human CRC stage II [90]. AGPAT2 association to cancer has been extensively reported in several human cancers and its inhibition has been explored as a potential cancer therapy [84,89,91,92,93]. AGPAT4 is upregulated in human cutaneous melanomas obtained from microarray datasets [94]. AGPAT6 expression changes are found to predict clear cell renal cell carcinoma (ccRCC) growth, together with another five metabolic enzymes [95]. AGPAT9 expression correlates with prostate cancer tumor stage [96] and colorectal adenocarcinoma [97]. AGPAT11 reflected the tumor grade in breast and cervical cancer tissues [98]. Finally, it is suggested that the increased levels of PA, derived from AGPAT11 elevation, activate Ras signaling, and consequently MAP kinase and PI3K/AKT survival pathways, in the aforementioned tumors [98].

The next step in the TG formation is performed by PAP, which removes the phosphate group from PA, giving rise to DG. Elevated levels of this enzyme isoform 1 (Lipin-1) have been recently linked to several cancers. Lipin-1 is upregulated not only in lung adenocarcinoma (LUAD) cell lines, but also in lung tumor tissues. Inhibiting this enzyme could constitute a good therapeutic strategy, since lipin-1 inhibition reduces cell viability in tumor cells, but not in non-transformed cells, and potentiates LUAD cells’ sensitivity to other chemotherapy treatments [99]. It was also detected in the upregulation in melanoma, fibrosarcoma, and breast cancer cell lines (TNBC) [100] and in half of high-grade prostate cancers [101]. 

Distinct from the former steps in the TG synthesis, in which the enzymes’ inhibition would lead to their phospholipid products signaling obstruction or membrane biogenesis actions blockade, the last step in TG formation constitutes a controversial point. DGAT esterifies DG and FA–CoA, giving rise to the TG molecule. DGAT activation in order to enhance storage would be a potential strategy for cancer prevention, since DGAT-1 overexpression reduces proliferation and invasiveness in human fibroblasts [102]. However, DGAT inhibition promotes the accumulation of its substrate, DG, with different signaling effects in cancer progression [103,104,105]. In fact, endogenous FA availability per se represents an oncogenic stimulus that could lead to tumor development [106]. However, in a recent report it has been described how prostate cancer cells, which are able to uptake and store lipids in LD, present elevated levels of DGAT isoform 1, and blocking the expression of both DGAT1 and the hydrolase ABHD5, implicated in LPA biosynthesis, results in growth inhibition [107]. What is also interesting is the presence of DGAT-1, but no DGAT-2, in a mesenchymal model of breast cancer cells [108].

Numerous studies have investigated the role of regulatory RNAs on lipids store formation. In the case of miRNAs, they might be directed to inhibit LD biogenesis. For example, Shu-hao Hsu et al. identified miR-122 as a direct interactor of *AGPAT*1, 3 and 9 isoforms, and DGAT1. miR-122 it abound in the liver regulating cholesterol metabolism, where its diminished expression leads to metastasis and poor prognosis in hepatocellular carcinoma (HCC) [27].

Moreover, circRNAs act as the sponge for miRNAs and function as competing endogenous RNAs for mRNAs. A previous established circRNA-miRNA-mRNA regulatory network has been identified underlying a metabolic effect. For example, the axis circRNA_021412/miR-1972/LPIN1, which was characterized by decreased level of circRNA_021412 and miR-1972 inhibiting LPIN1. LPIN1 downregulates long chain acyl-CoA synthetases’ (ACSLs) expression leading to hepatosteatosis, a potential complication for HCC [28].

Recent advances have revealed that lncRNAs play a crucial role in cell metabolism reprogramming in cancer cells, such us lipid metabolism [109]. The lncRNA SPRY4-IT1 expression is minimal in non-transformed human melanocytes but overexpressed in melanoma cells. It is able to directly bind LIPIN2 provoking the downregulation of DGAT2, triacylglycerol, fatty acyl chains, and acyl carnitine provoking cellular lipotoxicity [29]. 

Another important lncRNA, lnc-KDM5D-4, with a regulatory effect on lipin2, that in turn, lead to increased lipid droplet formation in HepG2 cells [30]. 

Overall, despite LD accumulation being reported in some specific cancers, its function in transformed cells is still not well identified [82]. Hence, when considering increasing LDs’ storage as a potential target for cancer treatment, we need to be cautious. It may be accompanied by LDs’ hydrolysis obstruction, explained in next section.

### 2.2. Lipid Droplets Hydrolysis

The second phase of the LDs’ status is the hydrolysis and release of their molecules (Figure 2A, lipids release). Three enzymes hydrolyze the TG molecule: triglyceride lipase (ATGL), hormone-sensitive lipase (HSL), and monoacylglycerol lipase (MAGL). Preventing the release of FAs from TG can be a strategy to reduce their availability in the cytoplasm.

ATGL is the rate-limiting enzyme in the TG hydrolysis cascade, hydrolyzing TGs into DG and FAs. Elevated ATGL (and, to an extent, HSL) activity may be a general feature of many cancer types as a strategy to obtain extra FA input. In fact, inhibitors of this enzyme resulted in the attenuation of cancer cell growth, such as in different lung carcinoma cell lines [110]. However, other studies reported the downregulation of ATGL protein expression to be linked with several malignant tumors, such as non-small cell lung cancers and pancreatic adenocarcinoma, as well as ovarian and breast tumors [111,112,113]. In these cases, the overexpression of ATGL or HSL by direct or indirect therapeutic means, could be detrimental for the patient, due to its association with cachexia, a multifactorial wasting syndrome in oncologic patients characterized by the uncontrolled loss of adipose tissue and muscle mass [114].

Finally, MAGL is able to decompose monoacylglycerol (MG) into free FAs, releasing the glycerol backbone. Upregulated expression of MAGL and enhanced de novo FA synthesis were observed in cell lines of several human cancers and primary tumors, including ovarian cancer, breast cancer, prostate cancer, and melanoma [115,116]. Interestingly, MAGL is overexpressed in androgen-independent prostate cancer cells, and chemical or genetic inhibition effects can be reversed by exogenous FA addition [117], raising the question of the importance of dietary regimes in cancer. 

Several investigations have explored the role of regulatory RNAs on the modulation of genes related to lipolysis. In this regard, two miRNAs have been studied in depth: miR-378 and miR-124. It is known that miR-378 improves adipocyte lipolysis via ATGL and HSL positive regulation [118,119], with special relevancy in human cancer cachexia [118]. MiR-124 also targets ATGL, leading to TG hydrolysis and promoting β-oxidation [120,121]. Furthermore, miR-425 is a positive regulator of both, ATGL and HSL [122], while miR-9 and miR-143 only positively regulate HSL [123]. Two negative miRNA regulators of lipolysis have been reported. They are miR-23b and miR-125 [124,125]. MiR-23b is implicated in ATGL and HSL regulation of thermogenesis [124] whereas miR-125 acts as a negative regulator of adipogenesis though FABP4 and ATGL [125]. 

However, none of these associations have been directly related to cancer development. In this sense, lncRNA-NEAT1 has been implicated in HCC development and lncRNA-SRA in the pre-malignant condition hepatic steatosis by regulating ATGL. LncRNA-NEAT1 positively regulates ATGL expression and alters lipolysis in HCC cells via ATGL. Particularly, ATGL and its products—DAG and FFA—promoted NEAT1-mediated HCC proliferation. Moreover, NEAT1 regulated ATGL expression via miR-124-3p [31]. No associations among other regulatory RNAs, cancer development, and HSL or MAGL have been reported to date.

In conclusion, there are again scarce data concerning lipids’ storage and release roles in cancer progression and their modulation through regulatory RNAs. Moreover, certain enzymes display dual roles, dependent on the tumor type, as well as LDs that show a controversial role in cancer. Further analysis is needed before considering them as potential targets for therapeutic strategies, as other routes could be more suitable when confronting cancer [4]. 

### 2.3. Fatty Acids Oxidation

Fatty acid oxidation (FAO) is mainly a repeated cycling for FA shortening (two carbons per cycle) producing NADH, FADH2, and acetyl-CoA in each cycle. NADH and FADH2, besides representing redox power, could enter the electron transport chain (ETC), therefore producing ATP. This process is principally carried out in energy-demanding tissues (heart and skeletal muscle) and in nutrient-providing organs (liver) [1].

It has long been thought that cancer cells override the utilization of metabolic intermediates for anabolic processes, which aim to construct building blocks for the cell (neutral/polar lipids, protein modifications). Therefore, enhancing catabolic routes such as FAs β-oxidation to limit their concentration levels could, in theory, be beneficial. Nevertheless, data from experiments testing this idea are contradictory. Cancer cells could require increased ATP production in different situations. In fact, greater evidence has been reported in recent years linking elevated levels of different enzymes to oxidation processes in cancer [1].

In FAO, the carnitine palmitoyltransferase system is essential for the transportation of FAs, especially long chain fatty acids previously activated as FA–CoA, from the cytoplasm into the mitochondria, to enter the β-oxidation route (Figure 2B). This transport system is composed by carnitine palmitoyltransferase I (CPTI), the carnitine acylcarnitine translocase (CACT), carnitine palmitoyltransferase II (CPTII), and the carnitine acetyltransferase (CrAT), which closes the carnitine cycle. The connection of this transport system with cancer has been extensively described by Melone et al. [126].

CPTI catalyzes the rate-limiting step of FAO, converting FA–CoA into the carnitine derivatives, and its overexpression in cancer has been broadly reviewed in Qu et al., explaining CPTI involvement in tumor neovascularization [127]. CPTI has three isoforms, all related to different types of tumors, and also reviewed in Melone et al. [126]. Recent studies reveal that targeting this enzyme sensitizes cancer to radiotherapy in nasopharyngeal carcinoma [128], or to metabolic therapy in human melanoma cells [129]. 

CACT catalyzes the exchange of carnitine/acetylcarnitine molecules. There are limited reports detailing the deregulated expression of CACT and cancer. An increase of both activity and expression of CACT is proposed as a hallmark in prostate cancer [36]. Besides, bladder cancer patients overexpress several carnitine-related enzymes, including CACT [130].

CPTII reconverts acylcarnitines to their respective long-chain acyl-CoAs. Little is known about CPTII deregulation in cancer, but some studies correlate its overexpression as an independent prognostic factor in CRC patients’ series [131]. Leukemia cells also display an upregulation of carnitine enzyme expression, including CPTII [132]. However, CPTII inactivity has been recently suggested to promote lipid accumulation-related hepatocarcinogenesis [133]; suggesting their distinctive tissue-dependent role and, therefore, CPTII overexpression, could be beneficial in certain situations.

CrAT, specifically for short-chain acyl-CoAs conversion, was also reported to be overexpressed in androgen-dependent and androgen-independent prostate tumor cells, and in prostate cancer patient’s biopsies, by Valentino et al. In line with this, a network comprising two members of the carnitine palmitoyltransferase system—CrAT, and CACT, together with CPT1A—makes prostate cancer cells more likely to utilize and oxidate FAs, contributing to the maintenance of their high metabolic plasticity [4,36]. 

Diverse studies have reported RNA regulation of genes belonging to the CPT system in a carcinogenic scenario. One of the seminal works analyzing molecular basis and pathophysiological consequences was performed in prostate cancer [36,37]. Authors identified miR-129-5p, miR-124-3p, and miR-378 as post-transcriptional modulators of, respectively, CACT, CPTIA, and CrAT genes in prostate cancer cells. They also studied human prostate cancer samples and detected deregulation of these miRNAs and genes in primary tumors [36,37]. 

In addition, it has been shown that metformin decreases lipid accumulation induced by high glucose levels in HCC cells through increasing the expression of CPTI and by downregulating the expression of miR-33b [32]. 

CPTI isoform C has also been implicated in tumor cell metabolism and cancer progression and it is regulated by miR-1291. CPTIC expression was indirectly and negatively correlated with miR-1291 levels in both breast and pancreatic cancer samples [38].

Numerous lncRNAs have been involved in the modulation of the expression of the CPTI in cancer. In gastric cancer cells, lncRNA-HCP5 was induced by mesenchymal stem cells. One study proposed that lncRNA-HCP5 targeted miR-3619-5p leading to the transactivation of CPTI and facilitated fatty acid oxidation [33]. Authors postulated that lncRNA-HCP5 could be a putative target for the improvement of chemotherapy efficacy in gastric cancer. LncRNA-NEAT1 is another potential therapeutic target of breast cancer. LncRNA-NEAT1 cooperates with miR-107 to modulate breast cancer growth and metastasis by targeting CPTI [34]. Furthermore, it has been reported that CPTI expression is modulated by lncRNA-LNMICC that promotes metastasis of cervical cancer and this regulation depends on FABP5 [35].

In general, the overexpression of enzymes belonging to the carnitine palmitoyltransferase system in cancer [126] suggests that FAs could serve as energy sources and metabolic fuel for tumor growth and proliferation through the mitochondrial oxidation pathway. However, controversial results exist, especially for CPTII [132,133], which complicate the establishment of therapeutic strategies based on these enzymes. Nevertheless, further studies over the entire system regulation could open a new avenue of cancer therapies [4].

### 2.4. Additional FAs Input

Fatty acid translocase (FAT), also named cluster of differentiation 36 (CD36), is a fatty acid receptor in charge of the identification and transport of extracellular FAs through the cell membrane (Figure 2C). CD36 has been demonstrated to uptake free fatty acids from the extracellular microenvironment to provide substrates for energy production and the synthesis of macromolecules supporting cell growth, proliferation, survival, and invasion in cancer [134]. Early studies about CD36 were focused on its role in angiogenesis and the promotion of atherosclerosis [135]. With the recognition of the relevance of fatty acid metabolism in cancer, the implication of CD36 in the initiation, and progression of the tumorigenic process has been highlighted. CD36 is found frequently dysregulated in various cancers affecting the tumor cell metabolism, survival, proliferation, and metastasis [136,137]. 

Regarding ncRNAs over this point, a role of miR-1254 as a tumor suppressor in oral squamous cell carcinoma mediated by the direct inhibition of the expression of CD36 has been reported [39].

Another family of enzymes in this group is Fatty-acid-binding proteins (FABPs), a small intracellular lipid transporters family with an affinity for long chain free fatty acids and other ligands. These enzymes transfer FAs from extracellular compartments, besides being involved in intracellular transport [138]. Although FABPs do not take part in de novo FA synthesis pathways per se, this family blockade needs to be taken into consideration, since some tumors gather lipids from their surroundings to potentiate de novo FA synthesis for their own benefit (Figure 2C) [8]. 

Distinct isoforms of this enzyme have been found to be upregulated in several tumors. For example, FABP4, mainly expressed in adipocytes and macrophages, may play a role in regulating hepatocarcinoma (HCC) progression in obesity scenarios [139]. Guaita-Esteruelas et al. recently reviewed the role of FABP4 and FABP5 derived from adipocytes in providing FAs to tumor cells. FABP4 and FABP5 are involved in tumorigenesis in different cancer types, including colorectal, prostate, oral, glioma, ovarian, and breast cancer [140]. However, controversial data are also found, such as those pointing to a negative correlation between FABP4 overexpression and cancer evolution [141]. Regarding FABP5, its overexpression is highly reported in cancers such as cervical, CRC, pancreatic, bladder cancer, or glioma, among others [140]. Another example is FABP7 and its implication in malignant glioma [142]. 

In this scenario, as previously described, lncRNA-LNMICC was reported to promote nodal metastasis of primary cervical cancer through the recruitment of the nuclear factor NPM1 to the FABP5 promoter; and interestingly, this pro-tumoral effect of LNMICC could be counteracted by miR-190 re-expression [35]. 

It is noteworthy that these enzymes perform not only intracellular but also extracellular actions. New studies point out their critical role as mediators of metabolic and inflammatory processes [143,144], and some specific isoforms are also involved in hormone control [145]. Therefore, regulatory RNAs targeting this family of enzymes would trigger an unexpected scale that should be assessed, which justifies further and deeper studies [4].

### 2.5. Fatty Acid Synthesis Enzymes

Finally, the main and most effective way to reduce FAs’ availability is to inhibit the enzymes responsible for their synthesis (Figure 2D). The linking point between glucose metabolism and de novo lipogenesis is citrate. From this point on, several enzymes could be altered for FA reduction. The citrate enters the Krebs cycle when it is located in the mitochondria. However, its translocation to the cytoplasm by mitochondrial citrate carrier (CIC) or SLC25A1, feeds de novo FA synthesis [146]. Limiting citrate translocation by inhibiting this carrier has been performed, resulting in anti-tumor activity. For example, CIC was reported as a novel target in p53 mutated tumors (lung and breast), and its inhibition strongly improves chemotherapy resistance and survival [147]. The chemotherapeutic resistance caused by CIC was also reported in non-small cell lung cancer (NSCLC), as well as a role in stemness maintenance [148]. Besides, CIC has been shown to be key in the maintenance of mitochondrial homeostasis in breast tumor cells [149], as well as being involved in a gene signature differentially expressed in persistently chemotherapy-resistant ovarian tumors [150]. Furthermore, it has been recently reported that CIC is also key for the location of citrate pools in cells, allowing CSCs self-renewal capability—enhancing mitochondrial respiration while diminishing the harmful effects of reactive oxygen species (ROS) in NSCLC. Although some groups are developing therapies to target this carrier, CIC inhibition could lead to citrate accumulation inside the mitochondria, feeding the TCA cycle and therefore exacerbating the Warburg effect, which could represent a handicap for its potential use as a therapy. 

Blocking the next enzyme in the route, ATP citrate lyase (ACLY, ACL, or ATPCL), has stronger effects than CIC [148]. ACLY catalyzes the conversion of the six-carbon citrate molecule to oxaloacetate and two-carbon acetyl-CoA. Previous studies found the expression level of ACLY fairly increased in tumors compared to normal cells, including CRC, broadly reviewed by Khwairakpam et al. [151]. Recently, ACLY was postulated in renal carcinoma (RCC) as a promising biomarker and a potential target for the evaluation of RCC treatment aggressiveness [152]. In pancreatic tumorigenesis, it has been reported that acetyl-CoA has a use for histone acetylation and, in the mevalonate pathway, promotes cell plasticity and proliferation, again suggesting the potentiality of this target in pancreatic cancer [153].

Nevertheless, inhibiting this enzyme would affect other pathways, since its product acetyl-CoA is a core central molecule, not only to FA synthesis but also to glucose, glutamine, and mevalonate pathways [154], as well as to protein acetylation reactions, controlling major cellular processes such as autophagy, energy metabolism, and mitosis [155,156]. Therefore, therapeutic targets against ACLY could be a double-edged sword, potentially with significant side effects [4].

In a recent work, it has been demonstrated that lncRNA-FLJ22763 significantly suppressed malignancy of gastric cancer cells and inhibited xenograft tumor growth by targeting the expression of ACLY. Moreover, FLJ22763 down-expression may be an independent prognostic factor in patients with GC [40]. MiR-126b has been described to suppress mesotheliomas by inhibiting ACLY and de novo synthesis of FAs [41]. MiR-22, by targeting the 3′-UTR of ACLY, inhibited the de novo lipogenesis in preclinical models of osteosarcoma, prostate, cervical, and lung cancers [42]. ACLY overexpression has been associated with poor prognosis in breast cancer, and miR-22 by targeting ACLY, has been proposed as a potential biomarker of poor prognosis in breast cancer [43]. On the contrary, miR-182, by targeting pyruvate dehydrogenase kinase 4 (PDK4), has been described to promote lung cancer. Interestingly, this effect is reversed after silencing ACLY, demonstrating the crucial requirement of ACLY in the protumoral effect of the miR-182-PDK4 axis [44]. MiR-133b suppressed gastric cancer cell proliferation, by targeting the 3´UTR of ACLY in a PPARγ-dependent manner [45]. Thus, all these data demonstrate that targeting ACLY represents a novel strategy for cancer treatment. 

Acetyl-CoA carboxylase (ACC) is a rate-limiting enzyme in de novo fatty acid synthesis. It is a biotin and ATP-dependent enzyme located in the endoplasmic reticulum (ER), which irreversibly performs the carboxylation of acetyl-CoA to form malonyl-CoA. There are two ACC isoforms designated as ACC1 (ACCa or ACACA) and ACC2 (ACCb or ACACB) with different metabolic roles. Malonyl-CoA synthesized by ACC1 is the substrate for FA synthesis, but, if synthesized by ACC2, is a CPT inhibitor, thus preventing FA degradation via β-oxidation [157]. ACC has been extensively documented in cancer development and progression [157,158], especially ACC1 and its role in energy-sensing mechanisms, susceptibility, and tumor development [159,160,161]. In recent years, new ways to target gene expression have been developed, such as in the case of ACC1, which was successfully downregulated by nanovesicles, evidencing the implication of this isoform in CRC progression [162]. Dual inhibition of ACC1 and ACC2 has been observed to target proliferation and de novo FAs synthesis in aggressive human glioblastoma cells [163]. Curiously, a recent study has shown that, despite cetuximab-mediated Warburg effect reduction, and AMPK activation with the subsequent ACC phosphorylation and inhibition, the following ACC overexpression was able to compensate the inhibition and promote survival [164]. In the majority of studies, the overexpression of FA synthesis enzymes such as ACC, along with its immediate downstream group of enzymes known as FASN, indicates tumor progression and cell malignancy [4,157,161,165].

Regarding ACC, scarce studies have demonstrated the implication of non-coding RNAs on its regulation. In breast cancer, miR-195, by targeting the 3′-UTR of ACC1, was demonstrated to inhibit cell proliferation, migration, and invasion, indicating that targeting ACC1 may be also an interesting strategy in breast cancer treatment [46].

Fatty acid synthase (FASN) is possibly the most studied enzyme related to FA metabolism and cancer. FASN catalyzes consecutive condensation reactions to form long-chain fatty acids from a molecule of acetyl-CoA and malonyl-CoA, mostly generating 16-carbon palmitate. FASN is associated with poor clinical outcomes, presents a universal upregulation, and is involved in the progression, maintenance, and enhancement of the malignant phenotype in most human cancers (prostate, ovarian, breast, endometrial, thyroid, colorectal, bladder, lung, thyroid, oral, tongue, esophageal, hepatocellular, pancreatic, and gastric carcinomas, etc.) [160,166,167,168,169,170] as well as sarcoma gastrointestinal stromal tumors [171]. Despite the conclusive evidence for FASN involvement in tumor metabolic reprogramming, the clinical scenario has remained uncompleted regarding first generation inhibitors, although it is improving with next-generation FASN inhibitors [172]. FASN inhibitor have also largely failed due to problems with rapid weight loss, with consequently poor clinical utility [4,173]. 

In this scenario long regulatory RNAs could help to gain insight into this complex point of FAs metabolism. The lncRNA-PVT1 has been described to be upregulated in osteosarcoma promoting migration and invasion through the regulation of the miR-195/FASN axis [47]. More specifically, lncRNA-PVT1 was demonstrated to act as a molecular sponge of miR-195 which correlated with the increased expression of FASN. On the contrary, silencing the expression of lncPVT1 in osteosarcomas, augmented the effect of miR-195 on the inhibition of FASN expression [48]. The lncRNA-HAGLR has been associated to poor prognosis in NSCLC by augmenting the expression of FASN [49].

In addition, several sncRNAs have been reported to regulate the expression of FASN in many types of cancer. MiR-15a and miR-16-1, through direct binding to the 3´UTR of FASN mRNA, inhibited the expression of FASN in breast cancer, reducing proliferation and invasiveness [50]. Moreover, in triple negative breast cancer (TNBC) cells, metformin treatment augmented the expression of miR-193b that, by targeting the 3´UTR of FASN, lead to the promotion of apoptosis [51]. In HCC cells, miR-1207-5p reduced cancer cell progression by diminishing the Akt/mTOR pathway and its downstream molecular target FASN [52]. In osteosarcoma and breast cancer cells, miR-195, by direct targeting the 3′-UTR of FASN, also reduced cell survival, proliferation, invasion, and metastasis [46,53]. MiR-320 inhibited tumor progression by targeting FASN in NSCLC [54]. Upregulation of circFARSA was demonstrated to promote invasiveness by sponging miR-330-5p and miR-326 and suppressing their inhibitory effects on FASN expression [55]. 

Finally, it is worth briefly mentioning the role of sterol regulatory element-binding protein (SREBP) in cancer metabolism, which is able to modify metabolic genes’ expression. SREBP is conceived as the master transcriptional regulator of FA synthesis [174] and represents gene expression control of the main lipid metabolism enzymes. Three isoforms—SREBP1a, SREBP1c and SREBP2—have been acknowledged in mammals with overlapping functions. In non-transformed cells, it maintains lipid homeostasis [175], regulating genes from the routes previously mentioned (ACLY, ACC, FAS, as well as SCD-1, and GPAT). In addition, a growing number of reports suggest SREBP implication in uncontrolled cancer cells’ proliferation. An enhanced expression of SREBP-1 has been detected in prostate cancer patients [176]. SREBP-1 is also associated with breast cancer cells’ migration and invasion, and its upregulation has been proposed as a poor prognostic marker in breast patients [177]. SREBP1, along with its target genes (ACC, FASN), is overexpressed in human multiform glioblastoma (EGFR mutated), and both genetic and pharmacological inhibition significantly block tumor-derived xenograft growth [178]. Apart from interacting with lipid metabolic enzymes, it has also been involved in glycolytic enzymes crosstalk. In this way, PKM2 stimulates SREBP-2, leading to hepatocellular cancer cell proliferation [179]. Recently, it has been reported that either SREBP1 or SREBP2 knockdown decrease FA levels in CRC. These results have been reported in mitochondrial respiration, glycolysis, and FA oxidation reduction in cancer cells, impairing spheroids’ tumor formation and reducing tumor-derived xenografts growth [180]. Hence, SREBP-1 inhibition in cancer cells, leading to FA synthesis genes’ downregulation, could represent a therapeutic window to prevent cancer cell proliferation [4]. 

Long non-coding RNAs or miRNAs could represent two tentative tools to this aim. LncRNA-HR1 was demonstrated to repress the SREBP1c promoter activity and FASN expression, decreasing lipid metabolism in the Huh7 hepatocarcinoma cell line [56]. Moreover, multiple miRNAs have also been shown to regulate the intracellular lipid content by modulating the expression of SREBPs. In HCC, miR-21 inhibited the expression of the p53 activator HBP1, diminishing the p53 tumor suppression activity, and augmenting the expression of *SREBP1c* which promoted lipid accumulation and hepatic tumorigenesis [57]. In prostate cancer cells, miR-185 and miR-342, was described to target SREBF1 and FASN inducing apoptosis [58]. In glioma cells, miR-132 repressed FASN expression indirectly by targeting SIRT1 mediated expression of SREBP1c, which induced apoptosis and inhibited cell invasion [59]. In a similar way, miR-449 suppressed lipid accumulation in hepatocarcinoma cell lines by decreasing the expression of *SIRT1* and its downstream target *SREBP1c* [60]. 

### 2.6. Fatty Acid Activation and Desaturation

#### 2.6.1. Acyl–CoA Synthetases 

Before an FA enters any metabolic pathway, it needs to be activated by the addition of a molecule of coenzyme A (CoA). These free FAs are converted to FA–CoA by acyl coenzyme A synthetase (ACS) (Figure 2E) [181]. This allows the FA to enter multiple physiological and metabolic routes, such as TG synthesis and cholesterol esters, retinal esters and phospholipids, β-oxidation (peroxisomes, mitochondria), ω-oxidation (ER), elongation and/or desaturation, signaling molecules, and protein acylation, among others [182]. The largest family of mammalian ACS is the long-chain acyl–CoA synthetases (ACSL) family, which catalyzes the formation of acyl–CoA from FA, with 12–20 carbon atoms chain lengths [183]. To date, five isoforms of the ACSL mammalian family have been identified: ACSL1, -3, -4, -5 and -6, which present different preferences regarding the chain length of their FA’s substrates [182].

The metabolic reprogramming has been widely reported on this metabolic point for various types of cancers. Previous reports associated patients with inflammatory bowel disease (IBD) with the over-expression of ACSL1 and ACSL5 in the terminal ileum and colon [184]. IBD represents a risk feature for developing CRC [185], suggesting the possible role of these isoforms in CRC initiation. A systematic analysis of the ACSL family members’ gene expression in various kinds of cancers has also been made [186]. This analysis concluded that ACSL1 was upregulated in CRC but diminished in lung and breast cancer, as well as in the brain, cervical, esophageal, head and neck, leukemia, liver, and sarcoma cancers. Chen et al. also analyzed the prognostic value of ACSL1 in cancer progression, relating breast cancer and CRC patients’ poorer survival rates with increased ACSL1 expression. 

ACSL3 has been reported to be overexpressed in lung cancer [187] and melanoma [186], but, in contrast, lower levels were found in prostate cancer tissues [188] or ovarian cancer [186]. From the systematic analysis previously mentioned, ACSL3 was overexpressed in head-neck and liver cancer but under expressed in CRC.

ACSL4 was previously associated with the colon carcinogenesis [186,189,190]; it was associated with tumor cell growth in liver cancer [191] and was described as an independent prognostic factor in patient tissue analysis [192]. Similarly, it has been implicated in cell growth, invasion, and hormonal resistance in prostate cancer cell lines [193], positively correlated with a specific subtype of breast cancer, and frequently downregulated in gastric cancer tissues [194]. ACSL4 was downregulated in tumor bladder, brain, leukemia, and lung cancer [186]. On the contrary, ACSL5 lower expression was observed in the CRC tissue, leading to longer, disease-free survival (DFS) [195]. High expression of ACSL5 predicted good prognosis in glioma [196], breast, ovarian, and lung tumors. ACSL6 was decreased in most forms of cancers, except for CRC, according to Chen’s analysis [186]. In addition, the expression of ACSL6 was downregulated in leukemia with good prognosis, emerging as a potential tumor suppressor gene in leukemia [4,186].

Due to its solid implication in cancer, this FA metabolic point needs to be taken into consideration for a deep posttranscriptional analysis. In this sense, several epigenetic processes have been observed regarding ACSL targeting in cancer. Firstly, specific miRNAs have been explored extensively in this line. This is the case of miR-205, able to decrease ACSL subunit 1 mRNA expression levels in hepatoma cells provoking a reduced lipogenesis [61]. This miRNA was also reported to directly downregulate another isoform of ACSL, ACSL4, also in hepatoma cells leading in this case to hepatic cholesterol accumulation [64].

Furthermore, rarely does a particular miRNA act as a specific biomarker or is potent enough to target cancer progression. Specially reviewed are miRNAs signatures. For example, the network miR-544a, miR-142, and miR-19b-1 were described as key controllers of the metabolic axis of activation and desaturation, ACSLs-1, -4 and SCD in colon cancer cells. This miRNA network is able to downregulate both protein levels and RNA of the ACSL/SCD pro-tumorigenic axis [66]. Another example of miRNAs signature affecting this point is in CRC, where ACSL6 appears to be controlled, among other 20 genes by a joined signature of 20 deregulated miRNAs. Specifically, let-7c, let-7e, miR-133a, miR-133b, miR-191-5p, and miR-222-3p were reported to affect these genes [68]. For instance, in a specific type of lung cancer (NSCLC) where ACSL5 appears differentially expressed, among the other 8 genes, it has been proven to be downregulated by a complex of 6 miRNAs (miR-149, miR-205, miR-375, miR-378, miR-422a, and miR-708) [67], where we can also find the previously mentioned miR-205. 

It is also remarkable that different regulatory RNAs can work as a network. For instance, two small, non-coding RNAs, a small nucleolar RNA (snoRNA) and the small nucleolar host gene 7 RNA (SNHG7), foster proliferation and migration of thyroid Cancer cells by sponging an miRNA, miR-449a, and therefore upregulating ACSL1 [62]. However, it exists that some snoRNAs are potent enough to act in a single manner like SNORD113-1, which operates as a tumor suppressor role in HCC, in a cohort of patients where ACSL4 is overexpressed [65]. 

Especially limited is the use of siRNAs as a therapy. Two siRNAs have been shown to be of great importance in mouse neuroblastoma, pU6-487i and pU6-586i, able to target ACSL6 and reduce cell proliferation [69]. 

The recent detection of lncRNAs controlling lipid metabolism opens a new avenue in fatty acid regulation, which can provide new targets for dyslipidemia. For example, the Highly Up-Regulated In Liver Cancer long non-coding RNA (LncRNA-HULC) is able to activate acyl-CoA synthetase subunit ACSL1, involving also in this process miR-9 and PPARA, contributing to the malignant development of hepatocellular carcinoma (HCC) [63]. 

At this point is important to briefly mention a type of regulated necrosis caspase-independent named ferroptosis. Cells die upon a lipid peroxidation iron-dependent. Regarding tumor cells, it was described that tumor cells evading other types of cell death are more sensitive to ferroptosis opening a major way for therapeutic exploitation [197,198]. ACSL4 is responsible for esterification of various FAs (such as arachidonic acid or adrenic acid) with CoA forming an Acyl-CoA which could suffer a ß-oxidation or underg0 PUFA biosynthesis, especially sensitive to lipid peroxidation and, therefore, ferroptosis [199]. Of special interest is the extensive review performed by Zhang and colleagues discussing about the crosstalk between noncoding RNAs and ferroptosis [200].

#### 2.6.2. Stearoyl-CoA Desaturase 

Stearoyl-CoA desaturase (SCD) is a rapidly degraded ER membrane protein (Figure 2E), downstream of ACSLs’ action. It presents a clear preference for palmitoyl-CoA (C16:0) and stearoyl-CoA (C18:0), producing palmitoleoyl-CoA (C16:1) and oleoyl-CoA (C18:0), respectively. The products of SCD desaturation also act as substrates for the synthesis of different lipids, such as phospholipids, diacylglycerols, triglycerides, cholesterol, and wax esters. There are two isoforms of SCD in human cells (SCD1 and SCD5). SCD1 gene is ubiquitously expressed, while SCD5 expression is limited to certain adult human tissues [201,202]. 

SCD represents the biosynthetic source of components for a plethora of lipids, such as the precursors oleic and palmitoleic acids, the most abundant monounsaturated fatty acids (MUFAs) with central roles in tumor progression, allowing cellular membrane biogenesis, energy storage, and cell signaling [203]. 

The ratio between saturated versus unsaturated fatty acids (SFA/MUFA) is crucial for cancer cells, since its alterations affect fluidity and protein dynamics. Several studies point to elevated MUFAs in blood cancer patients causing an increase in cancer episodes, poorer prognosis and a higher death rate in patients [204,205] (for review, see [206]). Likewise, over the years, SCD has been reported to have a determinant role in various types of tumor, including lung, breast, prostate, colon, kidney, thyroid, and lymphoma, and its inhibition has proven efficacy in suppressing different tumor aspects (reviewed in [203,206]). For instance, SCD regulates apoptosis in hepatocarcinoma [207] and prostate cells [208], increases hepatoma cells proliferation [209], and its inhibition reduces tumor cells’ survival in breast and prostate cancer [210]. 

Regarding CRC, SCD is able to promote tumor cell proliferation and inhibit apoptosis [211], besides augmenting metastasis through MUFA increased production [212]. In addition, SCD is involved in several gene signatures, evidencing that de novo fatty acid synthesis is increased in these tumors [90,213]. 

SCD1 is key for the control of the overall scenario of lipid synthesis in cancer cells [206], such as in ER stress, lipid-mediated cytotoxicity and, eventually, cell death [214], cell proliferation, survival, cell cycle progression, colony formation, and invasiveness, which leads to tumor formation and metastasis [206]. Therefore, being one of the masters of the overall scenario of lipid synthesis leads to a hard attention regarding posttranscriptional regulation as a potential therapeutic [4].

Among the most studied regulatory RNAs over this point we found miRNAs, both oncogenic and anti-oncogenic. CRC cell migration and invasion could be inhibited by miR-215 via downregulating SCD [70]. What are also remarkable are the oncogenic miR-221 and miR-222 by direct targeting of SCD5 in metastatic cell lines [75]. On the contrary, miR-600 low levels are implicated in WNT signaling through SCD1 activation and poor prognosis of breast tumors [71]. 

Interestingly, a circRNA comes from the SCD 3’UTR location but it does not affect the gene. The reported SCD-circRNA 2 is upregulated in HCC and para-carcinoma tissues and could serve as a biomarker of the disease [215]

SCD is also regulated by lncRNAs in cancer. For example, the lncRNA-UPAT together with the protein associated transcript UHRF1 up-regulate SCD-1 and Sprouty 4, a siRNA required for the survival of colon tumor cells [72]. Furthermore, in CRC, a comparative study between five CRC tissues and their paired normal tissues by lncRNA sequencing, found SCD deregulated among others, and six abnormally expressed lncRNAs (i.e., CTD-2256P15.4, RP4-785G19.5, RP11-229P13.23, RP11-731F5.2, CTD-2537I9.12 and MSTRG.17303). Although not known yet, the direct regulation of these lncRNAs over SCD must be a point of departure for future analysis on this point [216]. A class of lncRNA, the ultraconserved RNA uc.372, specifically suppresses miR-195/miR-4668 to release the inhibition of functional target genes such as SCD1, ACC, CD36, and FAS leading to lipid accumulation in HepG2 cells [73]. The snoRNA host gene 16 (SNHG16) is significantly up-regulated in 314 adenomas and all stages of CRC. SCD is down-regulated upon SNHG16 silencing [74].

## 3. Conclusions

Non-coding and regulatory RNAs are some of the constituents of epigenetic mechanisms which play strategic roles in several processes of cancer. From the literature search employed on this review, we can conclude that lncRNAs and miRNAs are the most studied ncRNAs, and due to this advanced research some of them are currently potential therapeutic targets employed as robust biomarkers for the diagnosis and prognosis of different cancers. However, the rest of ncRNAs (sncRNAs, snoRNAs, piwi-RNAs, rRNAs, tRNAs) are less studied though from the limited literature that exists we can also elucidate promising roles. In fact, the study of ncRNAs networks would probably shed more accurate results on the mechanisms of cancer dysregulation, more than individual ncRNAs processes. Besides, ncRNAs cooperate with other epigenetic mechanisms such as histone modifications and DNA methylation, that might be also taken into account. Therefore, the elucidation of the mechanism of ncRNAs in cancer processes requires an integral study. To this aim, the creation of RNA profiling by bioinformatics tools together with the launch of new non-coding RNA databases are vital for the comprehension of ncRNA regulation in cancer. 

## Figures and Tables

**Table 1:** Non-coding and regulatory RNAs regulating directly FA metabolism enzymes involved in cancer processes.

Gene	Regulatory RNA	Tumor Type/ Cell Type	Sense	Reference
**Lipid droplets formation**				
AGPAT (-1,-3,-9)	miR-122	HCC	-	[27]
DGAT-1	miR-122	HCC	-	[27]
Lipin1	circRNA_021412/ miR-1972	HepG2-based hepatic steatosis	- -	[28]
Lipin2/DGAT-2	LncRNA-SPRY4	ML	+	[29]
Lipin2	Lnc-KDM5D-4	HCC	+	[30]
**Lipid droplets hydrolysis**				
ATGL	lncRNA-NEAT1/ miR-124-3p	HCC	+ -	[31]
**Fatty acids oxidation**				
CPT1	miR-33b	HCC	-	[32]
CPT1	lncRNA-HCP5/ miR-3619-5p	GC	+ -	[33]
CPT1	lncRNA-NEAT1/ miR-107	BC	+ -	[34]
CPT1	lncRNA-LNMICC	CC	+	[35]
CPT1A	miR-124-3p	PrC	-	[36,37]
CPT1C	miR-1291	BC, PC	-	[38]
CACT	miR-129-5p	PrC	-	[36,37]
CrAT	miR-378	PrC	-	[36,37]
**Extra FAs input**				
CD36	miR-1254	OSCC	-	[39]
FABP5	LncRNA-LNMICC/ miR-190	CC	- +	[35]
**Fatty Acid Synthesis**				
ACLY	LncRNA-FLJ22763	GC	-	[40]
ACLY	miR-126b	Ms	-	[41]
ACLY	miR-22	OS, PrC, CC, LC	-	[42]
ACLY	miR-22	BC	-	[43]
ACLY	miR-182	LC	-	[44]
ACLY	miR-133b	GC	-	[45]
ACC1/2	miR-195	BC	-	[46]
FASN	LncRNA-PVT1/ miR-195	OS	+ -	[47,48]
FASN	LncRNA-HAGLR	NSCLC	+	[49]
FASN	miR-15a/miR-16b	BC	-	[50]
FASN	miR-193b	TNBC	-	[51]
FASN	miR-1207-5p	HCC	-	[52]
FASN	miR-195	OS, BC	-	[46,53]
FASN	miR-320	NSCLC	-	[54]
FASN	CircFARSA/ miR-330-5p-miR-326	NSCLC	+ -	[55]
SREBP1	LncRNA-HR1	HCC	-	[56]
SREBP1	miR-21	HCC	+	[57]
SREBP1	miR-185, miR-342	PrC	-	[58]
SREBP1c	miR-132	Gl	-	[59]
SREBP1	miR-449	HCC	-	[60]
**Fatty acid activation**				
ACSL1	miR-205	HCC	-	[61]
ACSL1	SNHG7- miR-449a	TC	+	[62]
ACSL1	LncRNA-HULC/ miR-9	HCC	+ -	[63]
ACSL4	miR-205	HCC	-	[64]
ACSL4	SNORD113-1	HCC	-	[65]
ACSL (-1; -4)/SCD	miR-544a, miR-142, and miR-19b-1	CRC	-	[66]
ACSL5	miR-149, miR-205, miR-375, miR-378, miR-422a and miR-708 (signature)	NSCLC	-	[67]
ACSL6	let-7c, let-7e, miR-133a, miR-133b, miR-191-5p and miR-222-3p (part of a 20 miRs signature)	CRC	-	[68]
ACSL6	pU6-487i /pU6-586i	NB	-	[69]
**Fatty acid desaturation**				
SCD-1	miR-215	CRC	-	[70]
SCD-1	miR-600	BC	-	[71]
SCD-1	LncRNA-UPAT	CRC	+	[72]
SCD-1	uc.372 miR-195/miR-4668	HepG2	+ -	[73]
SCD-1	SNHG16	CRC	+	[74]
SCD-5	miR-221/miR-222	ML	+	[75]

Tumor Type Abbreviations: BC: Breast Cancer; CC: Cervical Cancer; CRC: Colorectal cancer; GC: Gastric Cancer; GI: Gastrointestinal Cancer; HCC: Hepatocellular Carcinoma; LC: Lung Cancer; ML: Melanoma; Ms: Mesothelioma; NB: Neuroblastoma; NSCLC: Non-small Cell Lung Cancer; OS: Osteosarcoma; OSCC: Oral Squamous Cell Carcinoma; PC: Pancreatic Cancer; PrC: Prostate Cancer; TC: Thyroid Cancer, TNBC: Triple Negative Breast Cancer.
